# Characterization of the complete chloroplast genome of *Rhodiola sachalinensis* and comparative analysis with its congeneric plants

**DOI:** 10.1002/2211-5463.13854

**Published:** 2024-07-04

**Authors:** Tianqi Sun, Yuman Tang, Lei Zhou, Xu Qiao, Xuan Ma, Huaxia Qin, Yu Han, Chun Sui

**Affiliations:** ^1^ Institute of Medicinal Plant Development (IMPLAD), Chinese Academy of Medical Sciences & Peking Union Medical College (Key Laboratory of Bioactive Substances and Resources Utilization of Chinese Herbal Medicine, Ministry of Education & National Engineering Laboratory for Breeding of Endangered Medicinal Materials) Beijing China; ^2^ New Cicon Pharmaceutical Co., Ltd Urumqi China

**Keywords:** chloroplast genome, comparative analysis, phylogenetics, *Rhodiola* L.

## Abstract

*Rhodiola*, belonging to the Crassulaceae family, is a perennial herbaceous plant genus. There are about 90 *Rhodiola* species worldwide, some of which have been reported to have medicinal properties. *Rhodiola sachalinensis* is a perennial medicinal herb within this genus and, in the present study, its chloroplast genome was sequenced, assembled, annotated and compared with 24 other *Rhodiola* species. The results obtained show that the chloroplast genome of *R. sachalinensis* is 151 595 bp long and has a CG content of 37.7%. The inverted repeats (IR) region of the *Rhodiola* chloroplast genome is the most conserved region, with the main differences being observed in the *ycf1* and *ndhF* genes at the IRb‐small single copy boundary, and *rps19* and *trnH* genes at the IRa‐large single copy boundary. Phylogenetic analysis showed that *Rhodiola* species form two major clades, and species with recorded medicinal properties, clustered together in one branch except for *R. dumulosa*. Within the genus, *R. sachalinensis* is most closely related to *Rhodiola rosea*, although comparative analyses showed that only *R. sachalinensis* and *Rhodiola subopposita* contained the *psbZ* gene, which encodes a highly conserved protein subunit of the Photosystem II core complex. Overall, the present study contributes to the understanding of the chloroplast genome of *Rhodiola* species, and provides a theoretical basis for the study of their genetic diversity and possible use as medicinal plants.

## Graphical abstract

The chloroplast genome of *Rhodiola sachalinensis* was sequenced, assembled, annotated and compared with 24 other *Rhodiola* species. It had a length of 151 595 bp and a CG content of 37.7%. Within the *Rhodiola* genus, *R. sachalinensis* is most closely related to *Rhodiola rosea*, despite *R. sachalinensis* and *Rhodiola subopposita* being the only species with a unique *psbZ* gene.

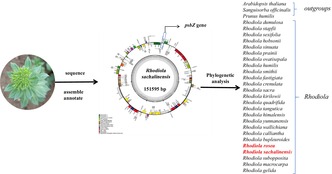

**Abbreviations**
CDS: coding sequences
IR: inverted repeats
LSC: large single copy
ML: maximum likelihood
SSC: small single copy
SSRs: simple sequence repeats

## Introduction

The genus of *Rhodiola* belongs to the family of Crassulaceae. There are approximately 90 species worldwide, and around 73 species, two subspecies and seven varieties found in China [1, 2]. Most *Rhodiola* plants grow on limestone at elevations between 3500 and 5000 m. They can adapt to harsh and variable natural environments, such as low oxygen levels, low temperatures, drought, strong winds, intense ultraviolet radiation and large diurnal temperature variations [3]. Many species within the genus of *Rhodiola* have been recognized for their pharmaceutical and health care value. Seven *Rhodiola* species, including *Rhodiola crenulata*, *Rhodiola sachalinensis*, *Rhodiola rosea*, *Rhodiola kirilowii*, *Rhodiola quadrifida*, *Rhodiola dumulosa* and *Rhodiola tangutica* have been clearly documented as the medicinal herbs [4, 5, 6].

At present, the primary active components of *Rhodiola* species can be categorized into nine groups, comprising over 60 compounds [7]. Among them, representative compounds include salidroside, tyrosol, rosavin and phenylpropanoids. *Rhodiola* species have demonstrated remarkable effects in combating fatigue [8], improving microcirculation [9], boosting immune function [10] and retarding the aging process. The medicinal plants of the genus of *Rhodiola* are utilized in the form of decoctions, drugs and extracts. Extracts from *R. rosea* and *R. crenulata* are currently manufactured in large quantities [11, 12, 13].

Chloroplasts are present in all green plants and certain autotrophic organisms, serving as important sites for photosynthesis [14]. Because of its moderate size, average rate of nucleotide substitution, significant differences in molecular evolutionary rates between coding and non‐coding regions, and a strong collinearity among chloroplast genomes of different plant groups, chloroplast genomes play a pivotal role in propelling the rapid development of the field of phylogenomics [15]. As of August 2023, the integration and collation of 29 069 chloroplast genomes from 16 435 species have been successfully contained by the comprehensive database Chloroplast Genome Information Resource (https://ngdc.cncb.ac.cn/cgir). At present, a total of 26 out of 90 *Rhodiola* species have undergone sequencing and their corresponding chloroplast genomes have been documented and deposited in the NCBI database. Chloroplasts genome of various *Rhodiola* species such as *R. kirilowii*, *Rhodiola sacra* and others have been reported [16, 17, 18, 19, 20].

*R. saccharinensis* is distributed in northeastern China, such as Jilin and Heilongjiang provinces. It has clear medicinal records. *R. saccharinensis* is included in the *Medicinal Herb Standards of Jilin Province* [21] and the *Traditional Chinese Medicinal Herb Standards of Zhejiang Province* [22]. The main active component of *R. saccharinensis* is salidroside, and it can reach 0.596% at the maturity of the herbs [23]. It has anti‐hypoxia, immune‐regulating, anti‐fatigue and excellent neuroprotective effects, showing great potential in the fields of plateau medicine and geriatric medicine [24]. Currently, *R. sachalinensis* is mainly studied for its chemical and pharmacological properties. However, its chloroplast genome has not been sequenced. The addition of *R. sachalinensis* chloroplast genomic information could expand the chloroplast genome library of the genus and facilitate the development and use of *R. sachalinensis*.

In the present study, we sequenced and assembled the chloroplast genome of *R. sachalinensis* using highthroughput sequencing technology. This resulted in a complete chloroplast genome sequence, contributing to the expansion of the chloroplast genome database for *Rhodiola* species. *Rhodiola* is a genus with complex origins and a wide variety of commercial medicinal materials, but some varieties are facing resource depletion [1, 25, 26]. Comparative analysis of the chloroplast genomes of *R. sachalinensis* and other 24 species was conducted, further investigating their phylogenetic positions and genetic relationships with closely related species, providing valuable information for study within the *Rhodiola* genus.

## Materials and methods

### Chloroplast DNA extraction and sequencing

#### Materials preparation

The rhizomes of *R. sachalinensis* were obtained from Nenjiang City, Heilongjiang Province, China (49.185766 N, 125.221192 E), cultivated and sampled in Haidian District, Beijing, China (39.959912 N, 116.298056 E). At the time of sampling, the experimental plant was in the growth stage with fully developed roots, stems and leaves. The plant specimen is currently stored in the National Engineering Laboratory for Endangered Medicinal Materials Breeding of the Institute of Medicinal Plant Development, Chinese Academy of Medical Sciences. The authenticity of the material was identified by Dr C. Sui. We first extracted DNA from the aerial parts of the plant using a Plant DNA Extraction Kit (Cat No. DP350‐03; Tiangen, Beijing, China). The quality of the DNA samples was then assessed using agarose gel electrophoresis. Subsequently, the Qubit 3.0 fluorometer was used for precise quantification, and DNA samples with a concentration above 1.5 μg were used for library construction.

#### Library construction, quality control and sequencing

DNA samples (0.2 μg) were used as input material for the DNA library preparations. The library construction was performed using the Rapid Plus DNA Lib Prep Kit (Cat No. RK20208; Illumina, San Diego, CA, USA). The manufacturer's recommended protocol was followed. Briefly, genomic DNA sample was fragmented by sonication to a size of about 350 bp. The DNA fragments were then end‐modified, A‐tailed and ligated to full‐length connectors suitable for Illumina sequencing, followed by PCR amplification. The PCR products were purified using AMPure XP system (Beckman Coulter, Brea, CA, USA). Subsequently, library quality was assessed on the Agilent 5400 system (Agilent, Santa Clara, CA, USA) and quantified by quantitative PCR (1.5 nm). Libraries meeting quality standards were mixed and sequenced on the Illumina platform of Novogene Bioinformatics Technology Co., Ltd (Beijing, China) under the PE150 strategy, based on the desired effective library concentration and data volume [27]. The original fluorescence image files obtained from Illumina platform were transformed to short reads (raw data) by base calling and these short reads were recorded in Fastq format [28], which contains sequence information and corresponding sequencing quality information. Basic statistical analysis was conducted on the quality of the raw reads using fastp, version 0.20.0 (https://fredhutch.github.io/easybuild-life-sciences/updates/2020-12-04-fastp/), and reads with adapter sequences, as well as low‐quality nucleotides, were discarded. In addition, polyG and polyX tails trimming was also performed. This ensured reliable downstream bioinformatics analysis.

### Chloroplast genome assembly and annotation

getorganelle, version 1.7.5 [29], was used with the default settings to assemble the chloroplast genome from the sequencing data, resulting in a circular chloroplast genome. cpgavas2 [30] was utilized for genome annotation of the chloroplast genome, and ogdraw [31] was used to visualize the chloroplast genome map. The tRNA genes in the chloroplast genome were annotated using trnascan‐se [32]. The rRNA genes in the chloroplast genome were annotated using blastn [33]. Finally, cpgview [34] and apollo [35] were employed for the annotation curation of the chloroplast genome.

### Acquisition of the chloroplast genome of some *Rhodiola* species

The published chloroplast genome sequences of 24 *Rhodiola* species were downloaded from NCBI, including *R. rosea* (MH410216.1), *Rhodiola calliantha* (NC_060675.1), *R. sacra* (NC_052735.1), *R. quadrifida* (NC_060680.1), *Rhodiola prainii* (MN794329.1), *R. tangutica* (NC_060605.1), *Rhodiola stapfii* (NC_060682.1), *Rhodiola himalensis* (NC_060677.1), *R. kirilowii* (NC_052736.1), *R. dumulosa* (MN794323.1), *Rhodiola smithii* (MN794331.1), *Rhodiola yunnanensis* (MN794332.1), *Rhodiola fastigiata* (MN794324.1), *Rhodiola gelida* (NC_063786.1), *Rhodiola humilis* (MN794326.1), *Rhodiola hobsonii* (MN794325.1), *Rhodiola bupleuroides* (NC_060674.1), *Rhodiola wallichiana* (NC_060683.1), *Rhodiola macrocarpa* (NC_060679.1), *R. crenulata* (MN794322.1), *Rhodiola subopposita* (OM161977.1), *Rhodiola sinuata* (NC_060681.1), *Rhodiola sexifolia* (NC_052737.1) and *Rhodiola ovatisepala* (MN794328.1). For species with multiple publicly released chloroplast genome sequences, those with higher credibility were selected for use after blasting the different sequences.

### Repeat sequences and simple sequence repeats (SSRs) analysis

reputer (https://bibiserv.cebitec.uni‐bielefeld.de/reputer) was used to identify forward, reverse, palindromic and complement repeat sequences. The minimum repeat size is limited to not < 30 basis points, the hamming distance value is 3 and other settings remain default [36]. tandem repeats finder (https://tandem.bu.edu/trf/trf.html) was used to detect tandem repeat sequences under default settings [37].

SSRs were identified using the online website MISA (https://webblast.ipk‐gatersleben.de/misa), including mono‐, di‐, tri‐, tetra‐, penta‐ and hexanucleotides with minimum numbers of 8, 4, 4, 3, 3 and 3, respectively, with parameter settings made with reference to Beier *et al*. [38].

### Comparative chloroplast genomes analysis

First, comparative analysis was conducted on three species, *R. sachalinensis*, *R. crenulata* and *R. rosea*, aiming to identify differentially genes among them. Subsequently, these selected genes were searched for in the chloroplast genomes of other *Rhodiola* species, aiming to identify genes that show significant differences among various species within the genus *Rhodiola*. The chloroplast genomes of *R. sachalinensis*, *R. crenulata* and *R. rosea* were compared using mvista, version 7 (https://genome.lbl.gov/vista/mvista/submit.shtml) to identify genes conservation. Then the inter‐specific differences of the sequences among *R. sachalinensis* and other 24 species are identified using mvista, version 7 [39, 40]. dnasp, version 6.0, was used for sliding window analysis to calculate nucleotide diversity (Pi) with parameter settings made with reference to Rozas *et al*. [41]. irscope (http://irscope.shinyapps.io/irapp) [42] was used to visualize the contraction and expansion of inverted repeats regions boundaries in these genomes.

### Phylogenetic analysis

The chloroplast genome sequences of all species were aligned using mafft, version 7.450 (https://mafft.cbrc.jp/alignment/software). Phylogenetic analysis was conducted using phylosuite (http://phylosuite.jushengwu.com) [43]. With the model plant *Arabidopsis thaliana*, as well as two species of *Rosaceae* including *Sanguisorba officinalis* and *Prunus humilis* as outgroups, a phylogenetic tree was constructed using the maximum likelihood (ML) based on the coding sequences (CDS) (Table S1) of chloroplast genomes from *R. sachalinensis* and other 24 species.

## Results

### General features of the chloroplast genome of *R. sachalinensis*

**Fig. 1:**
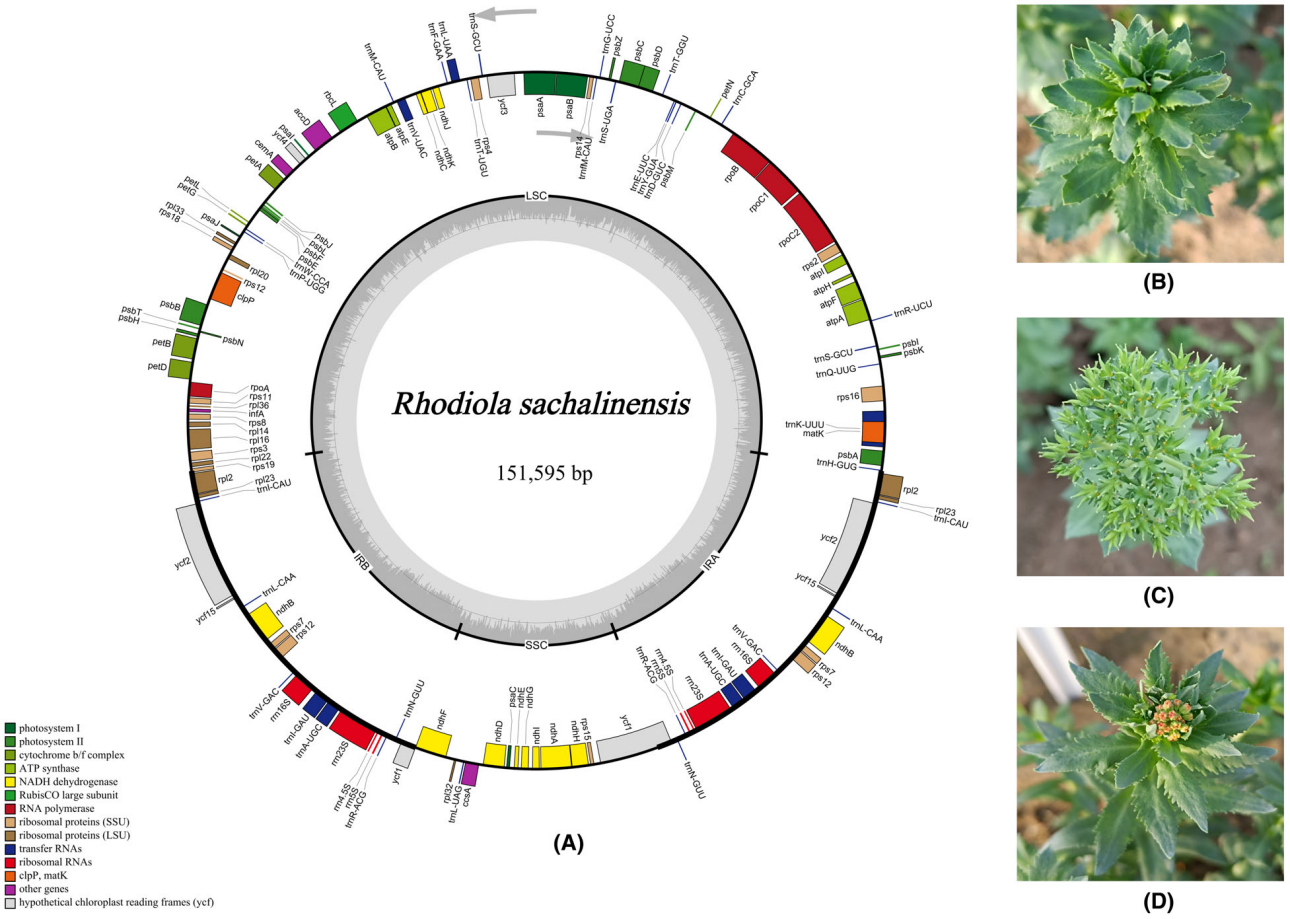
(A) Circular map of *Rhodiola sachalinensis* chloroplast genome created using cpgavas2. The circle map shows that the chloroplast genome of *R. sachalinensis* is a typical quadripartite structure with a total length of 151 595 bp. The genes inside and outside the circles are transcribed in clockwise and counter clockwise directions, respectively. The colors represent genes encoding different functional groups. The inner circle is divided into a dark gray area and a light gray area, representing the relative proportions of GC content and AT content in the genome, respectively. (B–D) Physical images of *R. sachalinensis* at different growth periods.

The chloroplast genome of *R. sachalinensis* showed a typical quadripartite structure. Its total length is 151 595 bp (Fig. 1), which is within the range of 150 286 bp (*R. smithii*) to 151 924 bp (*R. fastigiata*) in the chloroplast genome of *Rhodiola* species (Table 1). It is separated by a pair of 25 822 bp inverted repeats regions (IRa/b), with one large single copy region (LSC) (82793 bp) and one small single copy region (SSC) (17092 bp). The lengths of the IRa, IRb, LSC and SSC regions in *R. sachalinensis* are 33, 1, 176 and 50 bp longer than the average length of this segment in other 24 species, respectively (Table 1). The GC content is 37.7% in *R. sachalinensis*, which is in the average of the range of GC content of *R. sachalinensis* and other 24 species (Table S2). The chloroplast genome of *R. sachalinensis* was annotated with a total of 132 genes, including 88 protein‐coding genes, 36 tRNA genes and eight rRNA genes. *R. subopposita* and *R. ovatisepala* have the highest number of annotated genes, with a total of 134 genes each, whereas *R. tangutica* has the fewest number of genes, with a total of 128 genes. The original sequencing data and chloroplast genome sequence have been deposited into the NCBI database (OR555787).

**Table 1:** Basic features of the chloroplast genome from *Rhodiola sachalinensis*. Basic features of the chloroplast genome from *R. sachalinensis*. The A, T, G and C contents of the *R. sachalinensis* chloroplast genome sequence were statistically analyzed to obtain the contents of the different bases corresponding to each of the four regions of the chloroplast genome.

Region	A (%)	T (%)	C (%)	G (%)	GC (%)	Length (bp)
IRA	28.7	28.4	20.7	22.2	42.9	25 855
IRB	28.4	28.7	22.2	20.7	42.9	25 855
SSC	34.5	33.7	16.5	15.3	31.8	17 092
LSC	31.6	32.7	18.4	17.3	35.7	82 793
Total	30.9	31.4	19.2	18.5	37.7	151 595

### Genome annotation

Among the chloroplast genome of *R. sachalinensis*, there are nine CDS genes (*rps12*, *rps7*, *rpl23*, *rpl2*, *ndhB*, *atpF*, *ycf1*, *ycf2* and *ycf15*), eight tRNA genes (*trnA‐UGC*, *trnI‐CAU*, *trnI‐GAU*, *trnL‐CAA*, *trnN‐GUU*, *trnR‐ACG*, *trnS‐GCU* and *trnV‐GAC*) and four rRNA genes (*rrn16*, *rrn23*, *rrn4.5* and *rrn5*) that contain two repeat units (Table 2). The number of genes for rRNAs was eight for all 25 species, and the range of CDS gene numbers was between 84 abd 88. There were no significant differences in the total number of CDS genes, tRNA genes and rRNA genes. Comparison was made on the annotation of the chloroplast genomes of three species of *Rhodiola* (*R. sachalinensis*, *R. crenulata* and *R. rosea*). In these three species, the gene *psbZ* is present only in *R. sachalinensis*, the gene *trnS‐GGA* is unique to *R. crenulata* and the gene *trnS‐GCU* is contained only in *R. rosea*. The gene *lhbA* and *trnG‐GCC* are both absent from *R. sachalinensis*. Further statistical analysis revealed that, among *R. sachalinensis* and other 24 species, only *R. sachalinensis* and *R. subopposita* contained the *psbZ* gene, whereas neither of them contained the *lhbA* gene. The *psbZ* gene in other 23 species of *Rhodiola* was replaced by the *lhbA* gene.

**Table 2:** Gene list of three *Rhodiola* species. (2), the number of multi‐copy genes; ^, genes that only exist within *R. sachalinensis*; *, genes that only exist within *R. crenulata*; #, genes that only exist within *R. rosea*; *#, genes that exist within *R. crenulata* and *R. rosea*.

Function	Genes
Photosystem I	*psaA*, *psaB*, *psaC*, *psaI*, *psaJ*
Photosystem II	*psbA*, *psbB*, *psbC*, *psbD*, *psbE*, *psbF*, *psbH*, *psbI*, *psbJ*, *psbK*, *psbL*, *psbM*, *psbN*, *psbT*, *psbZ*^
Cytochrome	*petA*, *petB*, *petD*, *petG*, *petN*, *petL*
ATP synthase	*atpA*, *atpB*, *atpE*, *atpF* (2), *atpH*, *atpI*
NADH dehydrogenase	*ndhA*, *ndhB* (2), *ndhC*, *ndhD*, *ndhE*, *ndhF*, *ndhG*, *ndhH*, *ndhI*, *ndhJ*, *ndhK*
RubisCO	*rbcL*
RNA polymerase	*rpoA*, *rpoB*, *rpoC1*, *rpoC2*
Ribosomal proteins (SSU)	*rps2*, *rps3*, *rps4*, *rps7* (2), *rps8*, *rps11*, *rps12* (2), *rps14*, *rps15*, *rps16*, *rps18*, *rps19*
Ribosomal proteins (LSU)	*rpl2* (2), *rpl14*, *rpl16*, *rpl20*, *rpl22*, *rpl23* (2), *rpl32*, *rpl33*, *rpl36*
Transfer RNAs	*trnA‐UGC* (2), *trnC‐GCA*, *trnD‐GUC*, *trnE‐UUC*, *trnF‐GAA*, *trnfM‐CAU*, *trnG‐UCC*, trnG‐GCC*#, *trnH‐GUG*, *trnI‐CAU* (2), *trnI‐GAU* (2), *trnK‐UUU*, *trnL‐UAA*, *trnL‐CAA* (2), *trnL‐UAG*, *trnM‐CAU*, *trnN‐GUU* (2), *trnP‐UGG*, *trnQ‐UUG*, *trnR‐UCU*, *trnR‐ACG* (2), *trnS‐GCU* (2), trnS‐GGA*, *trnS‐GCU*#, *trnS‐UGA*, *trnT‐GGU*, *trnT‐UGU*, *trnV‐UAC*, *trnV‐GAC* (2), *trnW‐CCA*, *trnY‐GUA*
Ribosomal RNAs	*rrn4.5* (2), *rrn5* (2), *rrn16* (2), *rrn23* (2)
Clp P, matK	*matK*
Other genes	*accD*, *cemA*, *infA*, *ccsA*, *clpP*, lhbA*#
Hypothetical chloroplast reading frames	*ycf1* (2), *ycf2* (2), *ycf3*, *ycf4*, *ycf15* (2)

### Analysis of the repeat sequences and the SSRs

**Fig. 2:**
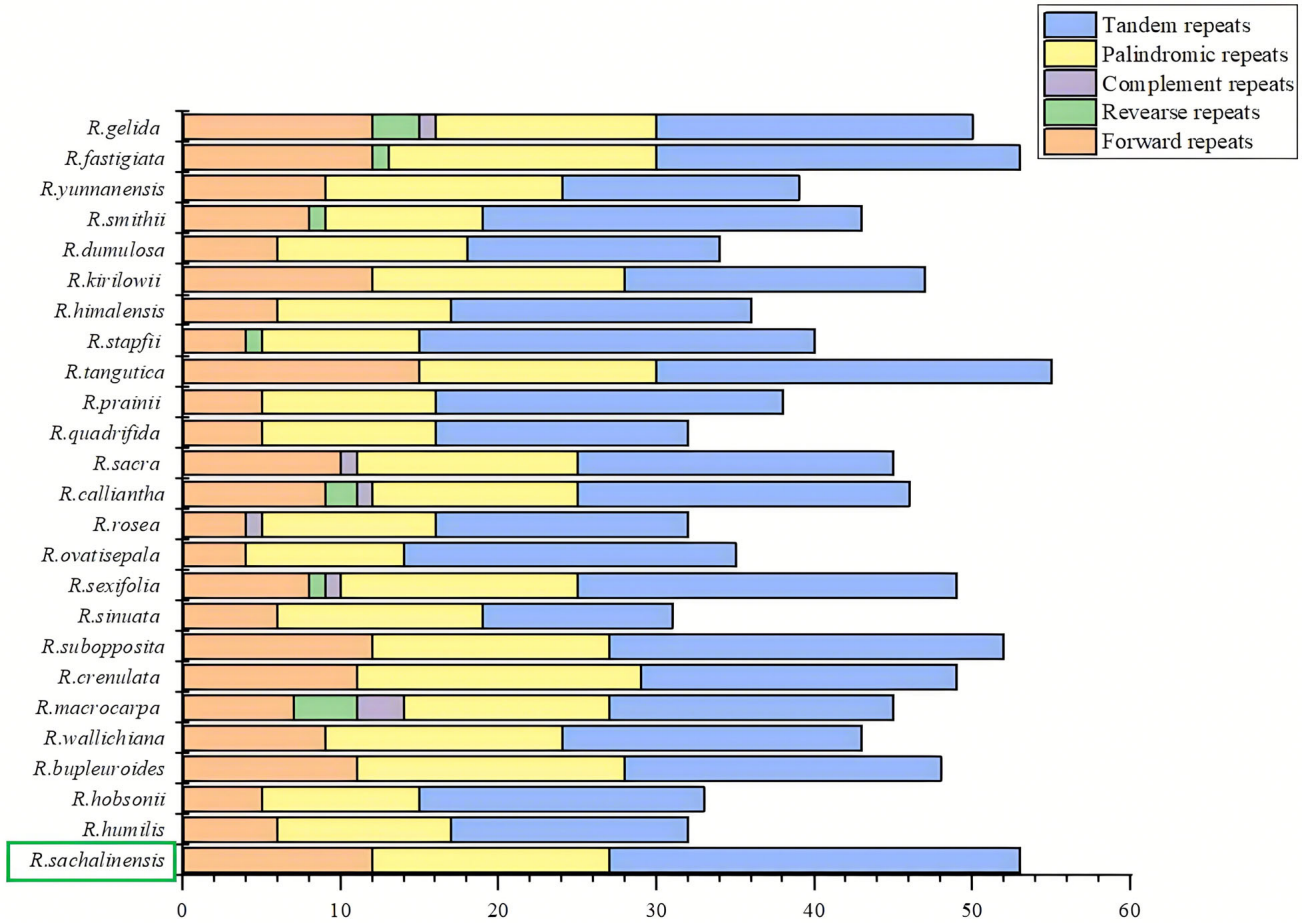
Numbers and types of repeat sequences in *Rhodiola sachalinensis* and the other 24 species chloroplast genomes. Forward, reverse, palindromic and complement repeat sequences were identified using reputer. Tandem repeat sequences were identified using tandem repeats finder. The detection of repeat sequences in the chloroplast genome of *R. sachalinensis* unveiled a total of 53 repeat sequences, comprising 12 forward repeat sequences, 15 palindromic repeat sequences and 26 tandem repeat sequences. This result is highlighted with a green box.

The detection of repeat sequences in the chloroplast genome of *R. sachalinensis* unveiled a total of 27 scattered repeat sequences, comprising 12 forward repeat sequences and 15 palindromic repeat sequences. However, no reverse or complement repeat sequences were detected. Additionally, the tandem repeat sequences exhibited by *R. sachalinensis* amounted to 26, with the majority being situated in the LSC (53.84%) and IRa region (19.23%). These tandem repeat sequences varied in length from 10 to 61 bp. Among *R. sachalinensis* and other 24 species, the range of dispersed repeat sequences in their chloroplast genomes is between 15 and 30, whereas the range of tandem repeat sequences is between 15 and 26. *R. macrocarpa* has the highest number of reverse repeat and complement repeat sequences, whereas *R. sachalinensis* has the highest number of tandem repeat sequences. Among *R. sachalinensis* and other 24 species, 16 species only contain three types of repeats, namely tandem repeats, palindromic repeats and forward repeats. Only four species, (*R. gelida*, *R. calliantha*, *R. sexifolia* and *R. macrocarpa*) have all five types of repeat sequences (Fig. 2).

**Fig. 3:**
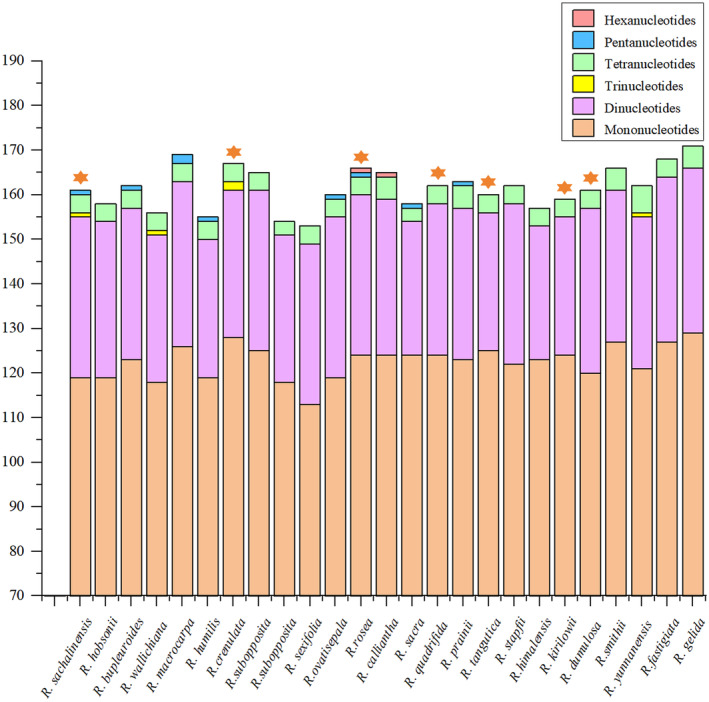
Numbers and types of SSRs in the *Rhodiola sachalinensis* and the other 24 species chloroplast genomes. Simple sequence repeat (SSR) loci were identified using the online website MISA. The total number of SSRs detected in the chloroplast genomes of 25 *Rhodiola* species ranges from 153 to 171, with *R. sachalinensis* having 161 SSRs loci. Among these SSRs, mononucleotide repeats are the most abundant (ranging from 60.0% to 69.7%). The result of the medicinal *Rhodiola* species are labeled with star symbols.

In *R. sachalinensis*, 161 SSRs loci were detected, containing five types of nucleotide repeats, with 119 mononucleotide repeats, 36 dinucleotide repeats, one trinucleotide repeat, one pentanucleotide repeat and four tetranucleotide repeats. The number of SSRs present in compound formation was 32. Among all the SSRs, the highest frequency is observed for A and T mononucleotide repeats, accounting for 72.67% of the total. This is followed by AT and TA dinucleotide repeats, which account for 13.67%. The total number of SSRs detected in the chloroplast genomes of *R. sachalinensis* and other 24 species ranges from 153 to 171, with *R. gelida* having the highest total number of SSRs. Among these SSRs, mononucleotide repeats are the most abundant (ranging from 60.0% to 69.7%), with the single nucleotide repeat units A and T, as well as the dinucleotide repeat units AT, GA, TA and TC, being shared by all individuals (Fig. 3). Specifically, *R. sachalinensis*, *R. wallichiana*, *R. crenulata* and *R. yunnanensis* exclusively possess trinucleotide repeats, whereas *R. rosea* and *R. calliantha* have hexanucleotide repeats.

### Comparative chloroplast genomes analysis

#### Sliding window analysis

**Fig. 4:**
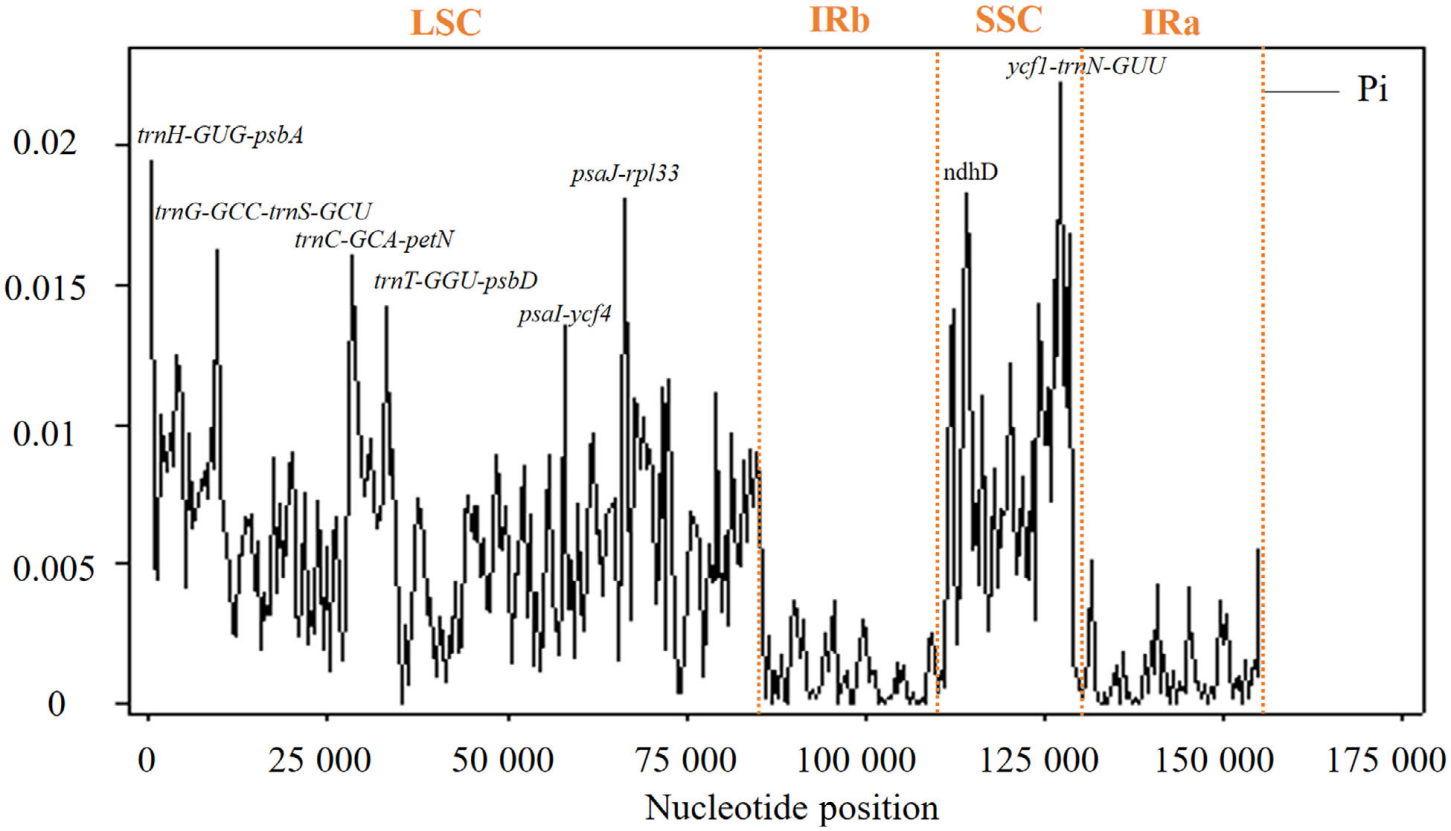
Nucleotide diversity (Pi) of chloroplast genomes of *Rhodiola sachalinensis* and the other 24 species. dnasp, version 6.0, was used to align and analyze *R. sachalinensis* and the other 24 species chloroplast genomes, with Pi values ranging from 0 to 0.02217. Except for the IRa/b regions, the Pi values in other regions are all > 0.005, indicating that the IR region is highly conserved. Eight relatively high variable regions with Pi values exceeding 0.0012 were labeled.

To quantify DNA polymorphism levels, we used dnasp, version 6.0, to align and analyze *R. sachalinensis* and other 24 species chloroplast genomes, with Pi values ranging from 0 to 0.02217. Eight relatively high variable regions with Pi values were detected exceeding 0.0012: *trnH‐GUG* – *psbA* (Pi = 0.01940), *trnS‐GCU* – *trnG‐GCC* (Pi = 0.001622), *trnC‐GCA* – *petN* (Pi = 0.01602), *trnT‐GGU* – *psbD* (Pi = 0.01421), *psaI* – *ycf4* (Pi = 0.01352), *psaJ* – *rpl33* (Pi = 0.01802), *ndhD* (Pi = 0.01825) and *ycf1* – *trnN‐GUU* (Pi = 0.02217). Among these highly variable regions, there are a total of six non‐coding regions and two coding regions. There are six highly variable regions located in the LSC region, which account for the highest proportion of variable regions (Fig. 4). Except for the IRa/b regions, the Pi values in other regions are all > 0.005.

#### Boundaries of IR

**Fig. 5:**
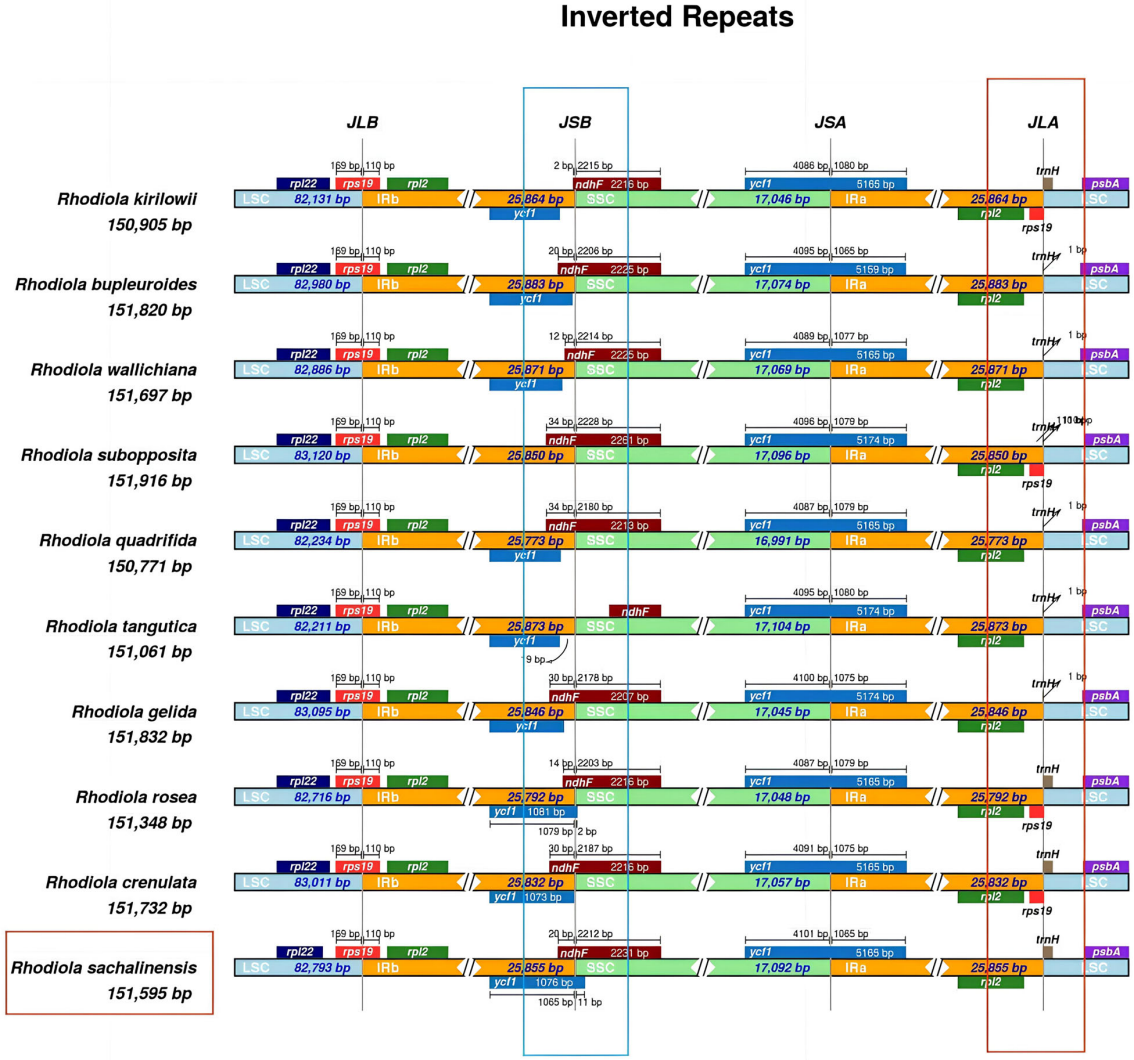
The chloroplast genomes of 10 species in the genus of *Rhodiola* were compared in terms of their boundary regions (LSC, SSC and IRs). irscope was used to visualize the contraction and expansion of inverted repeats regions boundaries in these genomes. Among them, the greatest differences were observed at the junction between IRb and SSC, as well as between IRa and LSC, which are indicated by boxes.

In some species of *Rhodiola* genus, the IR region of the chloroplast genome is the most conserved in terms of length and structure, but there is gene contraction or expansion at the junction of the IR with the LSC and SSC [44]. By comparing the same boundary of *R. sachalinensis* and other 24 species, most of the IR boundaries of *Rhodiola* did have obvious genetic differences, and 10 species with large differences were demonstrated (Fig. 5). The results showed differences in the *ycf1* and *ndhF* gene at the IRb and SSC boundaries. The *ycf1* gene in *R. sachalinensis* and *R. kirilowii* spans the IRb and SSC boundaries, whereas *R. subopposita* lacks the *ycf1* gene. The *ndhF* gene in *R. tangutica* is significantly contracted. There were significant differences in *rps19* and *trnH* gene at the IRa‐LSC boundary. At the boundary, the *rsp19* gene was absent in *R. sachalinensis*, *R. gelida*, *R. tangutica*, *R. quadrifida*, *R. wallichiana* and *R. bupleuroides*. Except for *R. sachalinensis*, the gene *trnH* was strongly contracted to 1 bp in the other five *Rhodiola* species.

#### Comparative analysis of chloroplast genomes

**Fig. 6:**
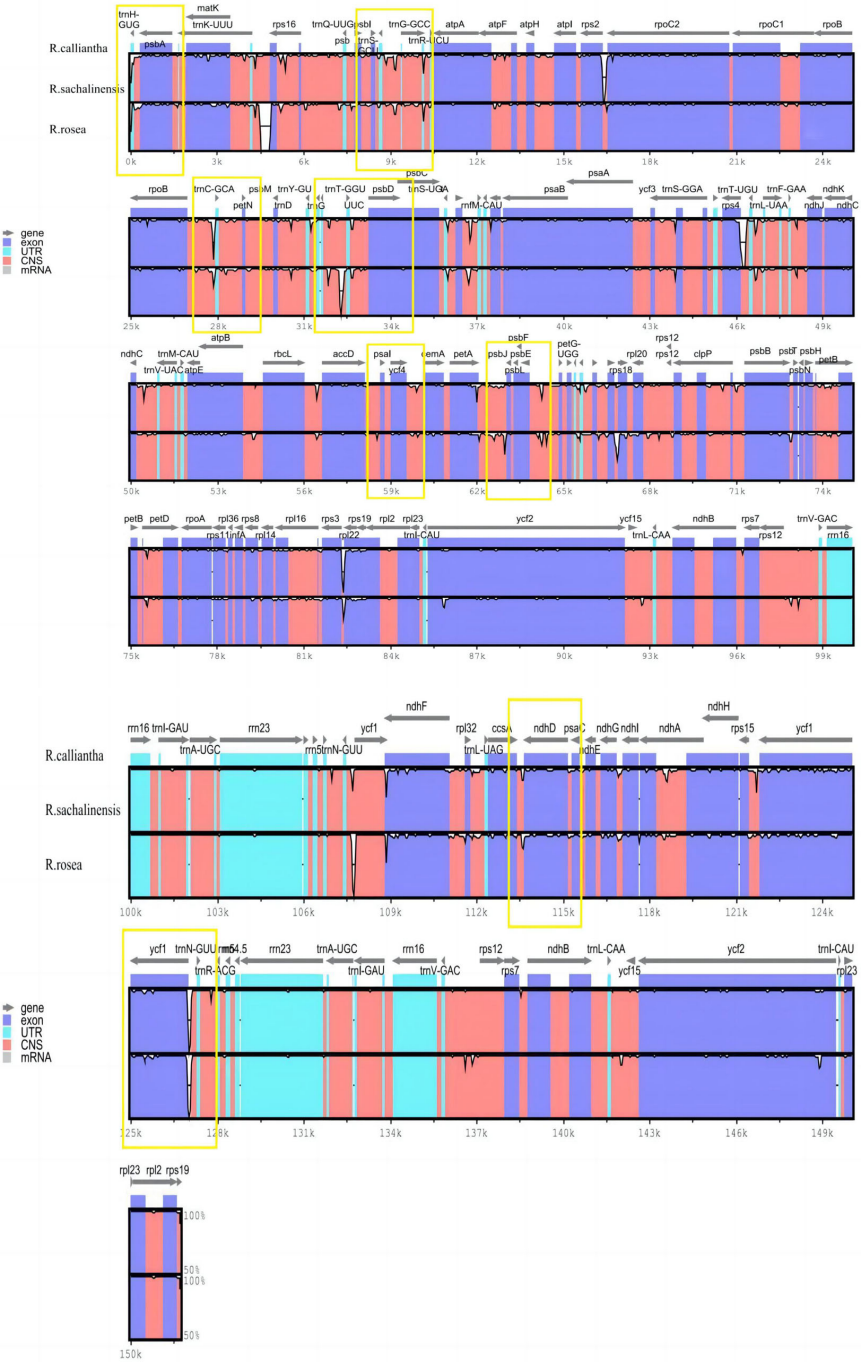
The comparative analysis of the chloroplast genomes of *R. crenulata*, *R. sachalinensis* and *R. rosea*. mvista, version 7, was used to find genes conservation from the chloroplast genomes of *R. sachalinensis*, *R. crenulata* and *R. rosea*. The arrows indicate the transcription direction, and different colors are used to distinguish different regions of the sequences. The comparative results of three species indicated that some intergenic regions, including *trnS*‐*GCU* – *trnG‐GCC*, *trnC*‐*GCA* – *petN*, *rpl32* – *trnL*‐*UAG*, *trnT‐GGU* – *psbD*, *psaI* – *ycf4*, and *psaJ* – *rpl33*, exhibited differences in distribution and length. The regions marked with yellow boxes indicate the significantly different areas.

The sequence variations of the chloroplast genomes of *R. sachalinensis* and other 24 species were analyzed, with *R. sachalinensis* being used as the reference annotation (Fig. S1). The results showed high sequence similarity in their chloroplast genomes. The IR region and coding region were more conserved than the single copy region and non‐coding region. The comparative results of three species of *R. crenulata*, *R. sachalinensis* and *R. rosea* were selected for the demonstration of gene differences, and some intergenic regions, including *trnS‐GCU* – *trnG‐GCC*, *trnC‐GCA* – *petN*, *rpl32* – *trnL‐UAG*, *trnT‐GGU* – *psbD*, *psaI* – *ycf4* and *psaJ* – *rpl33*, exhibited differences in distribution and length. A few protein coding genes such as *ycf1*, *ndhF*, *ndhA* and *ndhD* showed variations (Fig. 6). These results are consistent with the findings presented in the sliding window analysis.

### Phylogenetic analysis

**Fig. 7:**
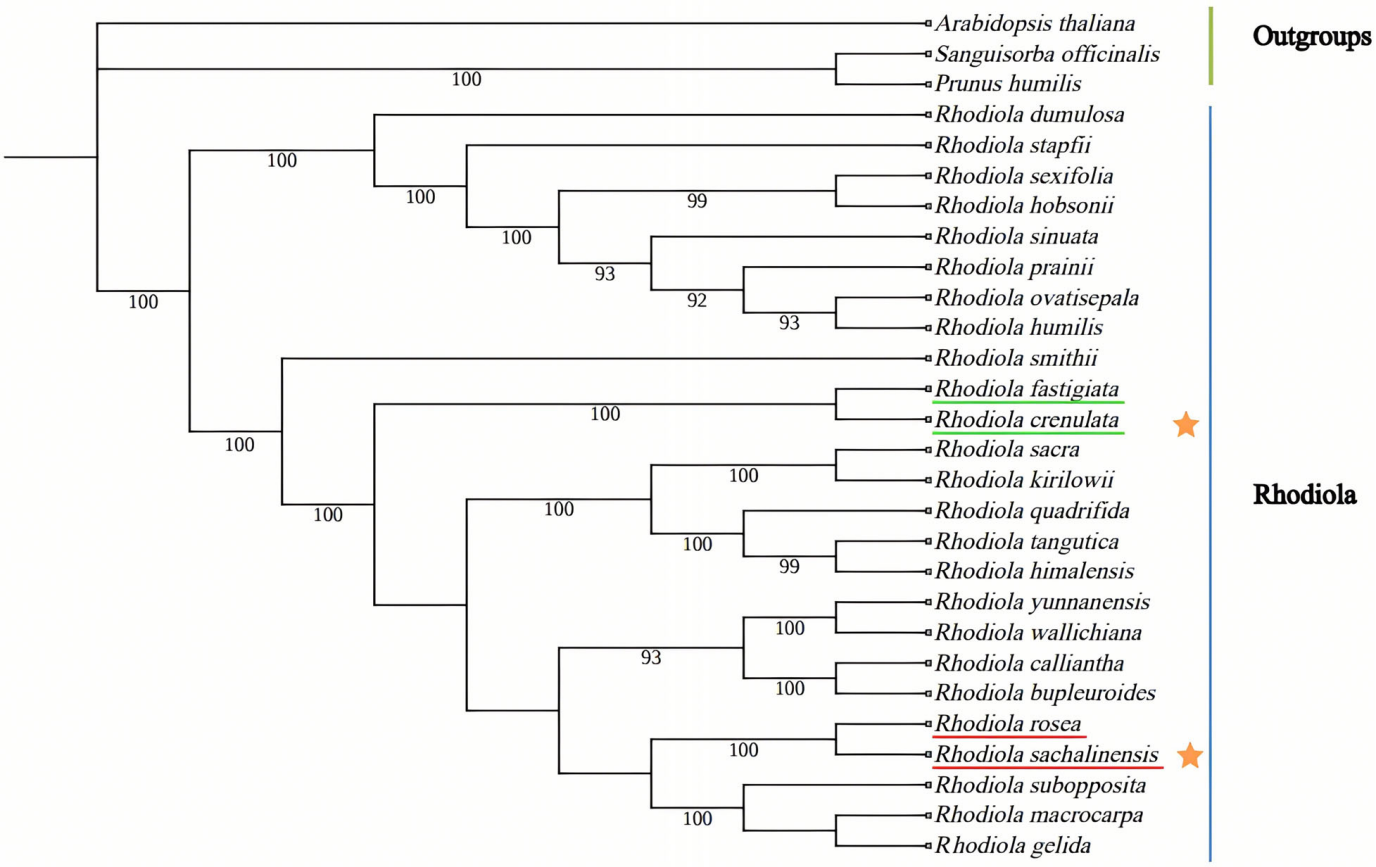
The phylogenetic tree of chloroplast genomes from *Rhodiola sachalinensis* and the other 24 species. The phylogenetic tree was constructed using the maximum likelihood (ML) based on the coding sequences (CDS) sequences of chloroplast genomes of the 25 species. The green color represents the selected outgroups, whereas the blue color represents the species from the genus of *Rhodiola*. *R. crenulata* and the sequenced *R. sachalinensis* are labeled with a star symbol. The support rate based on 1000 repetitions is displayed next to each branch node. The phylogenetic results indicate that 25 *Rhodiola* species are mainly divided into two clades. *R. sachalinensis* showed that it was most closely related to *R. rosea*.

The phylogenetic tree of the genus of *Rhodiola* was constructed with the model plant *A. thaliana*, and two species of Rosaceae including *S. officinalis* and *P. humilis* as outgroups. The group of *Rhodiola* formed two major branches with high support (100% bootstrap). Clade 1 includes eight *Rhodiola* species: *R. dumulosa*, *R. stapfii*, *R. sexifolia*, *R. hobsonii*, *R. sinuata*, *R. prainii*, *R. ovatisepala* and *R. humilis*. Clade 2 consists of the remaining 17 *Rhodiola* species. Phylogenetic analysis showed that the *Rhodiola* species with medicinal records clustered together in clade 2 except *R. dumulosa*. Regarding *R. crenulata*, which is the authentic source species of *Rhodiolae crenulatae radix et rhizoma* specified by the Chinese Pharmacopeia, the most closely related sister branch was *R. fastigiata*. However, *R. sachalinensis* showed that it was most closely related to *R. rosea* (Fig. 7).

## Discussion

### Genetic structure and molecular identification

The chloroplast genome of *R. sachalinensis* was sequenced for the first time and the results showed that it has a typical quadripartite structure. Unlike the overall deletion of an IR region, the IR region of *Rhodiola* species varies in an expansion and contraction that is common in angiosperms [45, 46, 47]. The total GC content of the chloroplast genome was shown to be 37.7%, which exceeded the median GC content of most angiosperms (35%), suggesting that the gene structure is relatively conserved [48, 49, 50, 51, 52]. The number of SSRs ranged from 153 to 171, with the highest number of A/T repeats in mononucleotide, thus confirming the generally accepted viewpoint that SSRs are mainly composed of short poly A and poly T [53, 54].

The genus of *Rhodiola* comprises a large number of species, exhibiting complex morphological variations among different species and even within individuals of the same species. Traditional morphological identification methods are insufficient to meet the demands of identification, especially in the medicinal field. Hence, research and analysis of chloroplast genomes can provide insights for subsequent species identification approaches. One of the applications of complete chloroplast genomes is their use as super DNA barcodes [55]. Zhang *et al*. [56] utilized the chloroplast genome to identify six *Dracaena* species. Wu *et al*. [57] constructed phylogenetic trees using ML and maximum parsimony methods, revealing high discriminatory power of the complete chloroplast genome for identifying *Fritillaria* species. Those studies indicate that complete chloroplast genomes can be directly applied for species identification. Furthermore, there are variations in the number, types, and compositions of SSRs loci among *R. sachalinensis* and other 24 species. For example, *R. sachalinensis*, *R. wallichiana*, *R. crenulata* and *R. yunnanensis* exclusively possess trinucleotide repeats, whereas *R. rosea* and *R. calliantha* have hexanucleotide repeats. These differences in SSRs loci can serve as potential markers for future identification of *Rhodiola* species. Additionally, the polymorphism of sequences was quantified through sequence comparisons using mvista and dnasp, version 6.0. Song *et al*. [58] utilized five variable regions in the chloroplast genomes of 18 rice varieties for identification purposes. Combined markers of the variable regions resulted in 100% varietal identification capacity. Park *et al*. [59] developed indel markers using indel sequences in *trnK*‐*trnQ* and *ycf1*‐*ndhF* from a chloroplast genome screen to accurately identify three closely related species: *Aconitum pseudolaeve*, *Aconitum longecassidatum* and *Aconitum barbatum*. In the present study, eight significantly different variable regions were identified among the chloroplast genomes of the *R. sachalinensis* and other 24 species. These regions exhibited high Pi values, indicating their potential for developing molecular markers to facilitate accurate identification and rational utilization of this genus.

### The sequence differences reflecting the inter‐specific diversity of *Rhodiola* species

Comparison of genes among *R. sachalinensis* and other 24 species showed no significant difference in the number of CDS genes, tRNA genes and rRNA genes, and most genes were conserved. Upon further analysis of the differential genes of the three *Rhodiola* species, it was found that only *R. sachalinensis* and *R. subopposita* contained the *psbZ* gene. In the remaining 23 species of *Rhodiola*, the expected gene *psbZ* position is replaced by the gene *lhbA*. *Sinocrassula indica* and *Sedum oryzifolium* of the Crassulaceae family also do not contain the *psbZ* gene. The PsbZ protein is a subunit of the photosystem II core complex and is highly conserved in most photosynthetic plants [60]. The PsbZ protein affects electron transfer and has a photoprotective function [61]. Studies by Swiatek *et al*. [62] and Ruf *et al*. [63] demonstrated the interaction between PsbZ protein and the photosystem II core complex and light‐harvesting complex II, as well as its critical role in non‐photochemical quenching under conditions of photoinhibition. Currently, there is limited research on the function of the *lhbA* gene, but partial evidence suggest that this gene is most likely to be lost in the evolution of *Camellia* plants [64]. Therefore, further investigations are warranted aiming to explore the underlying reasons for the occurrence of gene replacement from *psbZ* to *lhbA* in most *Rhodiola* species.

In addition, there are differences in the *ycf1* and *ndhF* genes at the IRb‐SSC boundaries in most *Rhodiola* species. The *ycf1* gene spans the IRb‐SSC boundary in *R. sachalinensis* and *R. kirilowii*, whereas the *ycf1* gene is missing in *R. subopposita*. There are also evident differences in the *rps19* and *trnH* genes at the IRa‐LSC boundary. Furthermore, it has been observed that some *Rhodiola* species have overlapping regions between *ycf1* and *ndhF* gene, with significant differences in the length of the overlap. For example, the overlap between *ycf1* and *ndhF* in *R. kirilowii* reaches a length of 87 bp. The *ycf1* gene encodes a protein involved in complex redox reactions in the photosynthetic electron transport chain [65]. The *ndhF* gene also encodes a protein involved in the assembly and function of the NADH dehydrogenase complex in the photosynthetic electron transport chain, playing a critical role in non‐cyclic photophosphorylation by converting light energy into chemical energy [66, 67]. Therefore, we can make the interesting speculation that, for *ycf1* and *ndhF*, comprising two important genes regulating photosynthesis, their overlap and the length of the overlap may have an impact on the transcription or translation of the proteins, thus influencing photosynthesis in different *Rhodiola* species.

### Phylogenetic analysis of the genus of *Rhodiola*

A phylogenetic tree of *R. sachalinensis* and other 24 species was constructed and the evolutionary tree reflected the proximity of kinship within the species. For example, *R. fastigiata* and *R. crenulata*, *R. sacra* and *R. kirilowii*, and *R. sachalinensis* and *R. rosea* are the sister branches with close affinity. *R. rosea* is widely used as an ingredient of dietary supplements in the Russian and American markets. Its close relative *R. sachalinensis* is also a medicinal species with clear therapeutic efficacy. This result suggests that closely related species are more likely to have similar potential for utilization. The current Chinese pharmacopeia specifies that the authentic source species of *Rhodiolae crenulatae radix et rhizoma* is *R. crenulata*. It is distributed on high‐altitude rocky shores ranging from 4000 to 5600 m above sea level. The natural habitat of *R. crenulata* is characterized by intense radiation and low oxygen levels. However, because of its high market demand, excessive human harvesting has resulted in a significant decline in the wild resource reserves in most regions, and some areas are on the verge of extinction [68]. The search for its alternative resources has been ongoing [69]. The development and utilization of *R. fastigiata*, as the closest relative of *R. crenulata*, is more worthy of further attention. However, Lynn Margulis suggested that the chloroplast genome is maternally inherited in the ‘endosymbiosis theory’ proposed in 1967 [70], which suggests that the conclusion of using the chloroplasts genome to judge genetic relationships has some limitations [71]. More accurate phylogenetic relationships still require comprehensive analysis of nuclear and organelle genes to obtain. Additionally, only 25 species of *Rhodiola* have been sequenced, and more chloroplast genomes of *Rhodiola* need to be sequenced in the future to obtain more comprehensive phylogenetic progression.

## Conclusions

In the present study, the chloroplast genome of *R. sachalinensis* was sequenced, assembled and annotated. The results showed that it is a typical quadripartite structure with a total length of 151 595 bp. The chloroplast genome contained 132 genes, 27 scattered repeat sequences and 161 SSRs loci. Comparative analysis of the chloroplast genomes of *R. sachalinensis* and other 24 species in the genus of *Rhodiola* showed that the chloroplast genomes were structurally conserved, but there were still contractions and expansions of genes at the boundaries of the IR region. Eight variable regions were identified among the chloroplast genome species, which provided reference information for developing chloroplast molecular markers in the genus of *Rhodiola*. The phylogenetic tree constructed using the ML method characterized the distance and proximity of the interspecific affinities and provided reasonable predictive information for the use of certain species. However, little of chloroplast genome of the genus was sequenced. With the enrichment of its chloroplast genome in the future, the genetic structure and evolution of *Rhodiola* species can be analyzed more systematically and *Rhodiola*. L species can be better utilized and developed.

## Conflicts of interest

Xuan Ma and Huaxia Qin have a commercial affiliation to New Cicon Pharmaceutical Co.

## Author contributions

STQ was responsible for the methodology and writing the original draft. STQ and SC were responsible for the methodology, writing the original draft and reviewing and editing. TYM was responsible for formal analysis. ZL was responsible for software. QX was responsible for validation. MX, QHX and HY were responsible for resources. SC was responsible for project administration. All authors have read and approved the final version of the manuscript submitted for publication.

## Supporting information


**Fig. S1.** Visualization and comparative analysis of 25 *Rhodiola* chloroplast genomes.
**Table S1.** The list of the CDS used to construct phylogenetic trees.
**Table S2.** Basic features of the chloroplast genomes from 25 *Rhodiola*.

## Data Availability

The data generated in this study can be obtained from the corresponding author upon reasonable request.
