# Supplementary material for: Characterization of the complete chloroplast genome of Rhodiola sachalinensis and comparative analysis with its congeneric plants
Source: FEBS Open Bio. 2024 Jul 4;14(8):1340–55. doi: 10.1002/2211-5463.13854 (PMC11301261; doi:10.1002/2211-5463.13854)
Supplement: Supplementary file 1 — Fig. S1. Visualization and comparative analysis of 25 Rhodiola chloroplast genomes. Table S1. The list of the CDS used to construct phylogenetic trees. Table S2. Basic features of the chloroplast genomes from 25 Rhodiola. [file FEB4-14-1340-s001.docx]

**Table S1** The list of the CDS genes to construct phylogenetic trees

| type | genes |
| --- | --- |
| Photosystem I | *psaA, psaB, psaC, psal, psaJ* |
| Photosystem II | *psbA, psbB, psbC, psbD, psbE, psbF, psbH, psbl, psbJ, psbK, psbL, psbM, psbN, psbT, psbZ* |
| Cytochrome | *petA, petB, petD, petG, petL, petN* |
| ATP synthase | *atpA, atpB, atpE, atpF, atpH, atpl* |
| NADH dehydrogenase | *ndhA, ndhB, ndhB_copy2, ndhC, ndhD, ndhE, ndhF, ndhG, ndhH, ndhl, ndhJ, ndhK* |
| RubisCO | *rbcL* |
| RNA polymerase | *rpoA, rpoB, rpoC1, rpoC2* |
| Ribosomal proteins (LSU) | *rpl2, rpl2_copy2, rpl14, rpl16, rpl20, rpl22, rpl23, rpl23_copy2, rpl32, rpl33, rpl36* |
| Ribosomal proteins (SSU) | *rps2, rps3, rps4, rps7, rps7_copy2, rps8, rps11, rps12, rps12_copy2, rps14, rps15, rps16, rps18, rps19, rps19_copy2* |
| Clp P, matK | *matK,* |
| Other genes | *IhbA , infA , clpP1 , clpP , cemA , ccsA , pbf1, accD* |
| Hypothetical chloroplast reading frames | *ycf1, ycf1_copy2, ycf2, ycf2_copy2, ycf3, ycf4, ycf15* |

|  |  | Genome Size (bp) | | | |  | AT% | Number of Genes | | | |
| --- | --- | --- | --- | --- | --- | --- | --- | --- | --- | --- | --- |
| Species | LSC | SSC | IRA | IRB | Total | GC% |  | CDS | tRNAs | rRNAs | Gene |
| *R. rosea* | 82716 | 17048 | 25792 | 25792 | 151348 | 37.7 | 62.3 | 85 | 37 | 8 | 134 |
| *R. calliantha* | 83073 | 17066 | 25584 | 25584 | 151307 | 37.6 | 62.4 | 85 | 37 | 8 | 134 |
| *R. sacra* | 82161 | 17034 | 25873 | 25873 | 150941 | 37.8 | 62.2 | 84 | 36 | 8 | 132 |
| *R. quadrifida* | 82234 | 16991 | 25773 | 25773 | 150771 | 37.8 | 62.2 | 84 | 37 | 8 | 123 |
| *R. prainii* | 82953 | 17053 | 25794 | 25794 | 151594 | 37.7 | 62.3 | 85 | 37 | 8 | 134 |
| *R. tangutica* | 82211 | 17104 | 25873 | 25873 | 151061 | 37.8 | 62.2 | 84 | 37 | 8 | 123 |
| *R. stapfii* | 82658 | 17100 | 25811 | 25811 | 151380 | 37.7 | 62.3 | 85 | 37 | 8 | 134 |
| *R. himalensis* | 82261 | 16994 | 25812 | 25812 | 150879 | 37.8 | 62.2 | 85 | 37 | 8 | 134 |
| *R. kirilowii* | 82131 | 17046 | 25864 | 25864 | 150905 | 37.8 | 62.2 | 84 | 36 | 8 | 132 |
| *R. dumulosa* | 82873 | 17009 | 25850 | 25850 | 151582 | 37.8 | 62.2 | 85 | 37 | 8 | 134 |
| *R. smithii* | 81651 | 17019 | 25808 | 25808 | 150286 | 37.8 | 62.2 | 85 | 37 | 8 | 134 |
| *R. yunnanensis* | 82561 | 17008 | 25844 | 25844 | 151257 | 37.8 | 62.2 | 85 | 37 | 8 | 134 |
| *R. fastigiata* | 83110 | 17054 | 25880 | 25880 | 151924 | 37.7 | 62.3 | 85 | 37 | 8 | 134 |
| *R. gelida* | 83095 | 17045 | 25846 | 25846 | 151832 | 37.7 | 62.3 | 84 | 37 | 8 | 123 |
| *R. humilis* | 82415 | 16784 | 25790 | 25790 | 150779 | 37.8 | 62.2 | 85 | 37 | 8 | 134 |
| *R. hobsonii* | 81998 | 17077 | 25783 | 25783 | 150641 | 37.8 | 62.2 | 85 | 37 | 8 | 134 |
| *R. bupleuroides* | 82980 | 17074 | 25883 | 25883 | 151820 | 37.7 | 62.3 | 84 | 37 | 8 | 123 |
| *R. wallichiana* | 82886 | 17069 | 25871 | 25871 | 151697 | 37.7 | 62.3 | 84 | 37 | 8 | 123 |
| *R. macrocarpa* | 82654 | 17080 | 25788 | 25788 | 151310 | 37.8 | 62.2 | 85 | 37 | 8 | 134 |
| *R. crenulata* | 83011 | 17057 | 25832 | 25832 | 151732 | 37.7 | 62.3 | 85 | 37 | 8 | 134 |
| *R. subopposita* | 83120 | 17096 | 25850 | 25850 | 151916 | 37.7 | 62.3 | 88 | 37 | 8 | 134 |
| *R. sachalinensis* | 82793 | 17092 | 25855 | 25822 | 151595 | 37.7 | 62.3 | 88 | 36 | 8 | 132 |
| *R. sinuata* | 82708 | 17043 | 25799 | 25799 | 151349 | 37.8 | 62.2 | 85 | 37 | 8 | 134 |
| *R. sexifolia* | 82830 | 17021 | 25877 | 25877 | 151605 | 37.8 | 62.2 | 84 | 36 | 8 | 132 |
| *R. ovatisepala* | 82348 | 17089 | 25818 | 25818 | 151073 | 37.7 | 62.3 | 85 | 37 | 8 | 134 |

**Table S2** Basic features of the Chloroplast genomes from 25 *Rhodiola*

**
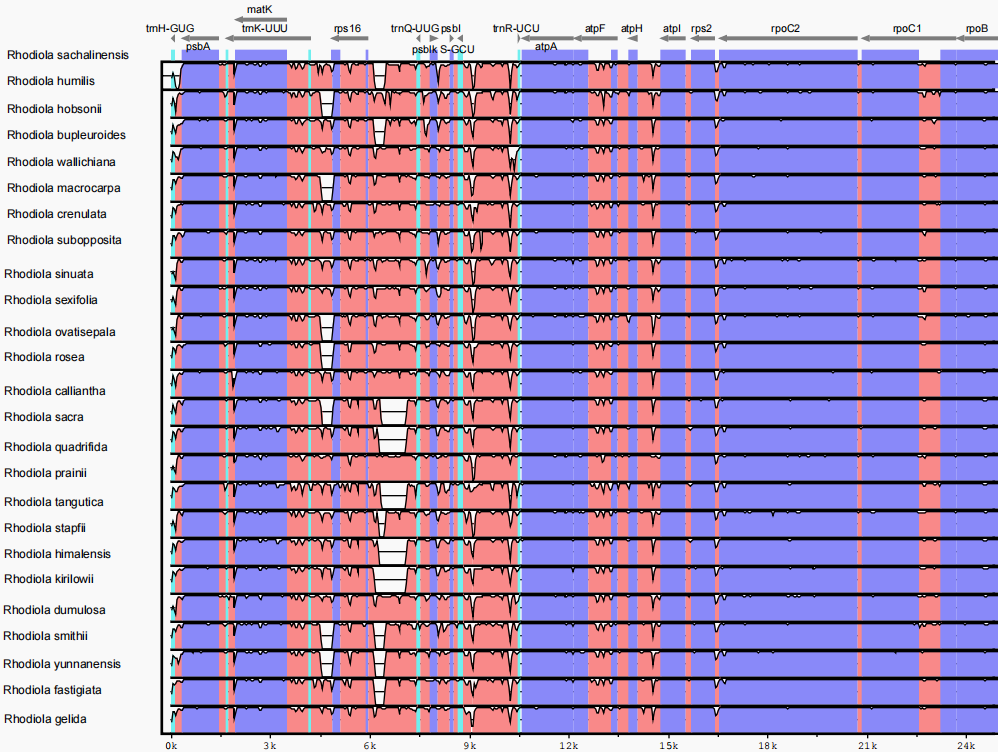

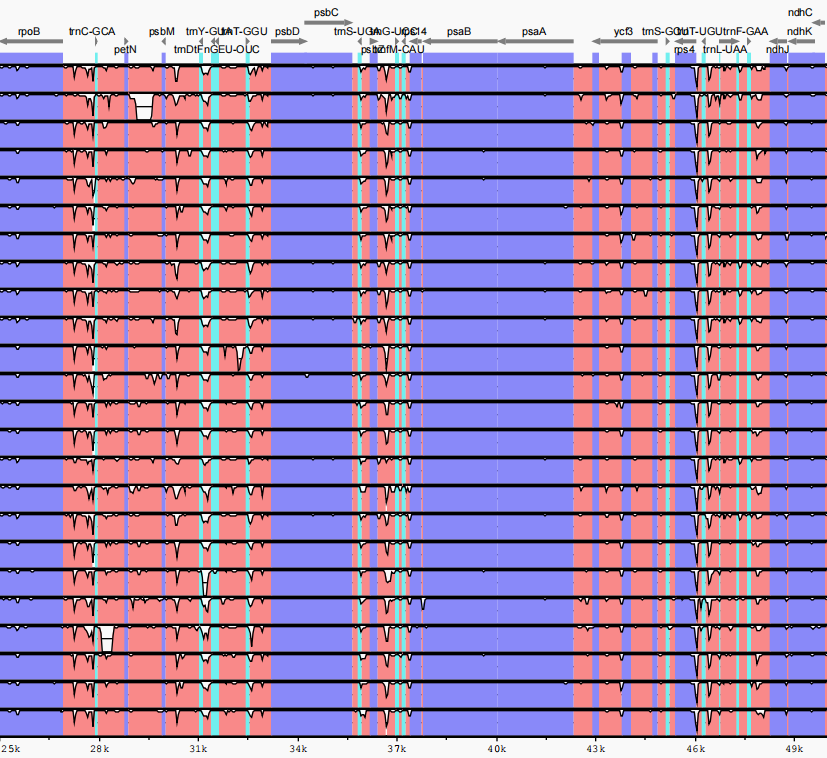

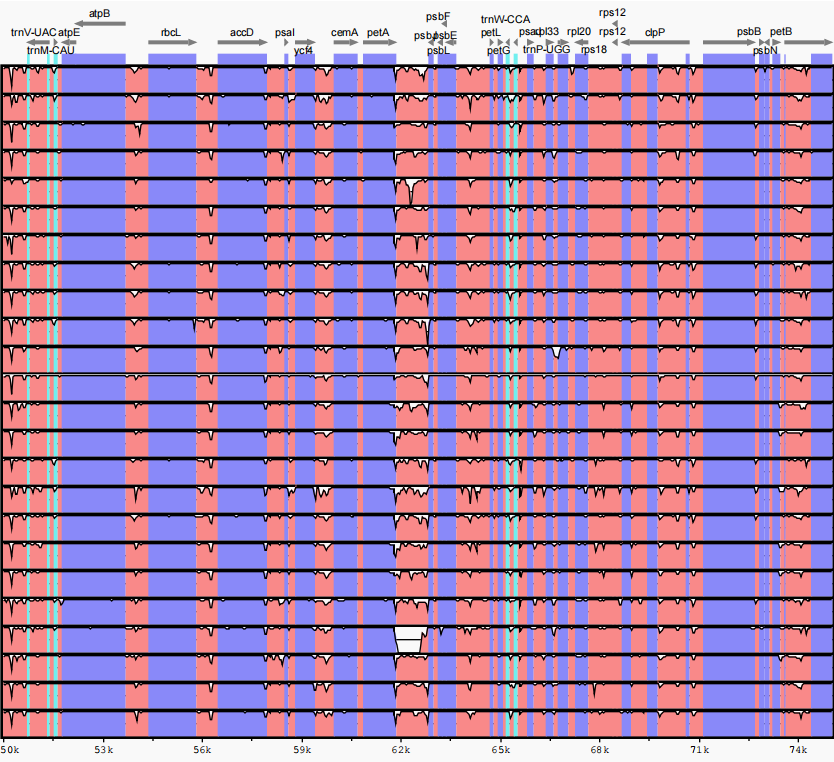

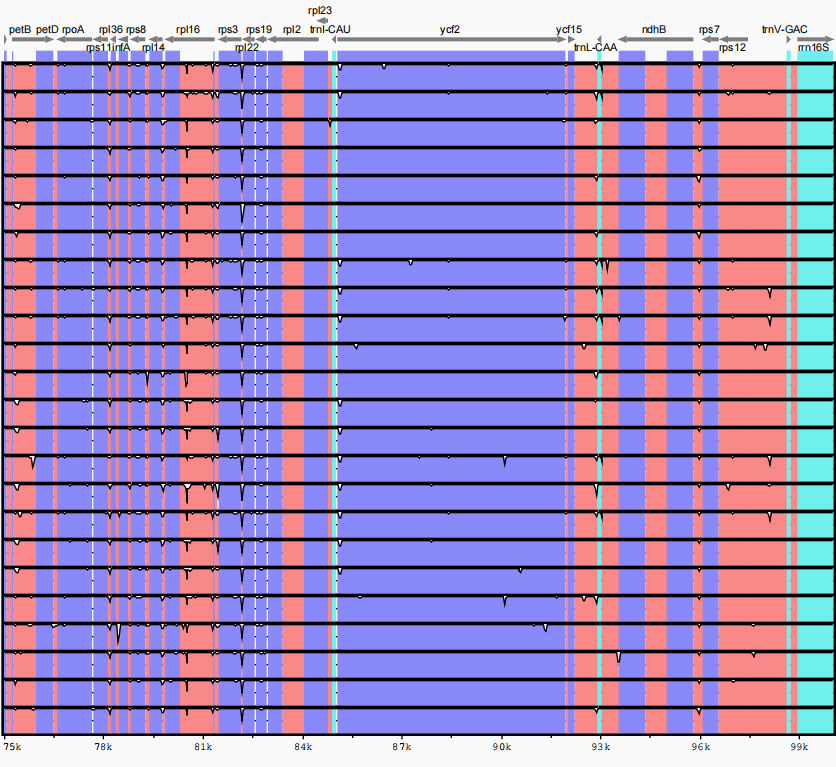

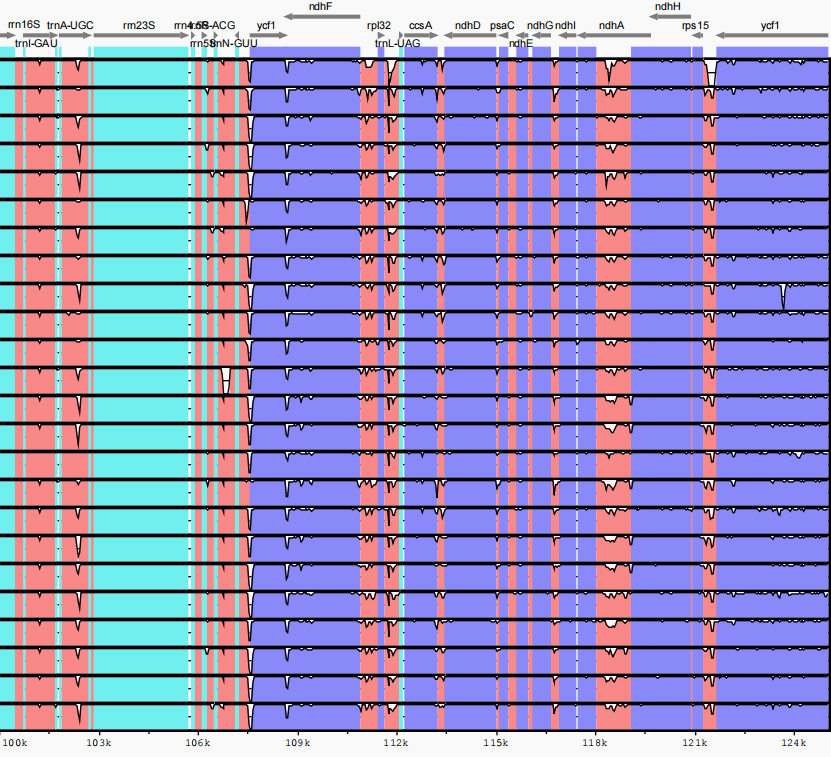

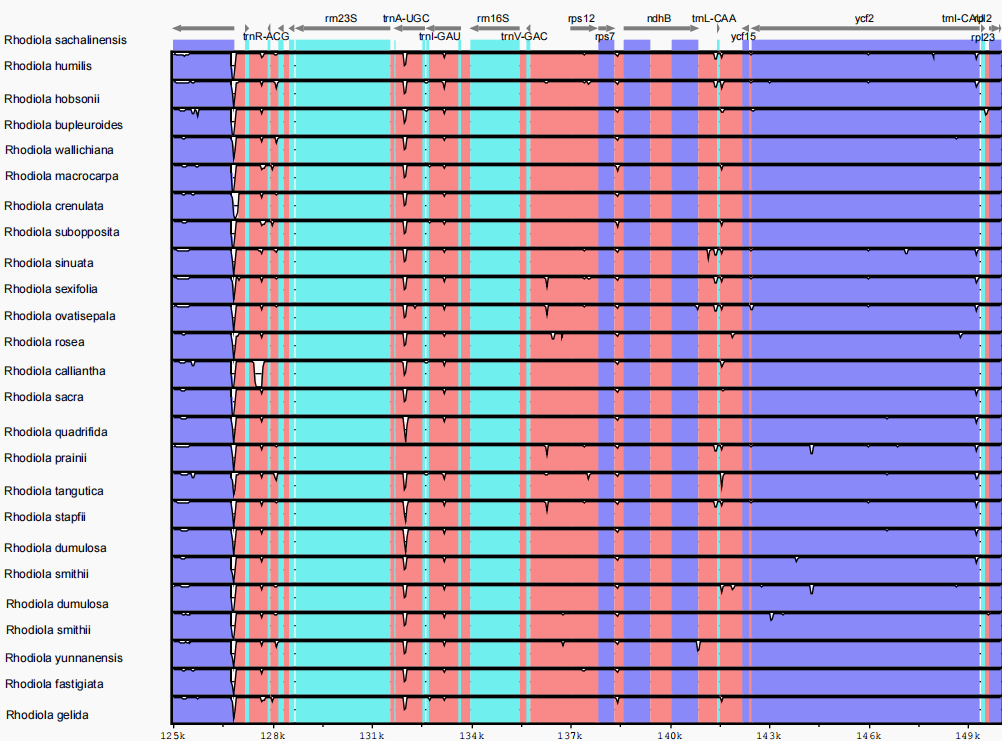

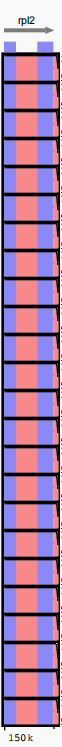
**

**Figure S1** Visualization and comparative analysis of 25 *Rhodiola* chloroplast genomes.
